# Household transmission investigation: Design, reporting and critical appraisal

**DOI:** 10.1111/irv.13165

**Published:** 2023-06-15

**Authors:** David J. Price, Violeta Spirkoska, Adrian J. Marcato, Niamh Meagher, James E. Fielding, Amalia Karahalios, Isabel Bergeri, Hannah Lewis, Marta Valenciano, Richard Pebody, Jodie McVernon, Juan‐Pablo Villanueva‐Cabezas

**Affiliations:** ^1^ Department of Infectious Diseases, The University of Melbourne Peter Doherty Institute for Infection and Immunity Melbourne Victoria Australia; ^2^ Centre for Epidemiology & Biostatistics, Melbourne School of Population & Global Health The University of Melbourne Melbourne Victoria Australia; ^3^ Victorian Infectious Diseases Reference Laboratory Epidemiology Unit, Royal Melbourne Hospital Peter Doherty Institute for Infection and Immunity Melbourne Victoria Australia; ^4^ World Health Organization Geneva Switzerland; ^5^ Epiconcept Paris France; ^6^ World Health Organization Regional Office for Europe Copenhagen Denmark; ^7^ The Nossal Institute for Global Health The University of Melbourne Melbourne Victoria Australia

**Keywords:** COVID‐19, epidemiology, emerging infectious diseases, pandemic preparedness, public health, influenza

## Abstract

**Background:**

Household transmission investigations (HHTIs) contribute timely epidemiologic knowledge in response to emerging pathogens. HHTIs conducted in the context of the COVID‐19 pandemic in 2020‐21 reported variable methodological approaches, producing epidemiological estimates that vary in meaning, precision and accuracy. Because specific tools to assist with the optimal design and critical appraisal of HHTIs are not available, the aggregation and pooling of inferences from HHTIs to inform policy and interventions may be challenging.

**Methods:**

In this manuscript, we discuss key aspects of the HHTI design, provide recommendations for the reporting of these studies and propose an appraisal tool that contributes to the optimal design and critical appraisal of HHTIs.

**Results:**

The appraisal tool consists of 12 questions that explore 10 aspects of HHTIs and can be answered ‘yes’, ‘no’ or ‘unclear’. We provide an example of the use of this tool in the context of a systematic review that aimed to quantify the household secondary attack rate from HHTIs.

**Conclusion:**

We seek to fill a gap in the epidemiologic literature and contribute to standardised HHTI approaches across settings to achieve richer and more informative datasets.

## 1 INTRODUCTION

Following the emergence of Severe Acute Respiratory Syndrome Coronavirus‐2 (SARS‐CoV‐2) and the subsequent Coronavirus disease 2019 (COVID‐19) pandemic, the World Health Organization (WHO) published a suite of investigation protocols collectively known as *The Unity Studies*.^1^ These protocols build upon *the CONSISE protocols for standardised research of influenza* ^2^ and other generic protocols^3^ to rapidly generate evidence‐based knowledge for action in response to emerging respiratory pathogens.^4^

Consistent application of a study protocol can facilitate reliable comparison and aggregation of data at the regional and global level.^4^ *The Unity Studies* include a protocol for household transmission investigations (HHTIs)^5^ that standardises data collection and analysis^6^ to rapidly determine critical epidemiological and clinical parameters of novel respiratory pathogens upon their emergence.^5^ Specifically, *The Unity Studies'* HHTI protocol provides guidelines for the targeted investigation of a defined population that may not readily mix with the community, facilitating the collection of rich epidemiological, virological and serological data. Coupled with appropriate analytics, these data inform the: (i) proportion of asymptomatic cases; (ii) incubation period, duration of infectiousness and detectable shedding; (iii) serial interval; (iv) reproduction number; (v) clinical risk factors and clinical course and severity of disease; (vi) high‐risk population subgroups; (vii) secondary infection rate and secondary clinical attack rate; and (viii) patterns of healthcare‐seeking.^5^

Rigorous and timely evidence generation during a public health emergency is challenging. Further, as stated in the Unity Studies HHTI protocol, investigators may need to adapt HHTIs to local circumstances—including public health mandates, laboratory and health system capacity, financial resources, sociopolitical context, and culture‐specific household practices.^5^ Such adaptations lead to heterogeneity in implementation.^7^, ^8^ For example, many HHTIs for COVID‐19 conducted throughout 2020 and 2021^8^, ^9^ were adapted to local context but not clearly reported.^8^ As a result, the collection, sharing, aggregation, analysis and reporting of epidemiological and biological data may vary in meaning, precision, and accuracy across settings. Several tools to assess the quality of epidemiologic studies have been applied or adapted to HHTIs,^10^, ^11^ but these may not adequately capture the complexities of investigations conducted during epidemics.

Here, we discuss considerations for the design of HHTIs, provide recommendations for their reporting and present a checklist for their critical appraisal.

## 2 THE HHTI DESIGN

**FIGURE 1:**
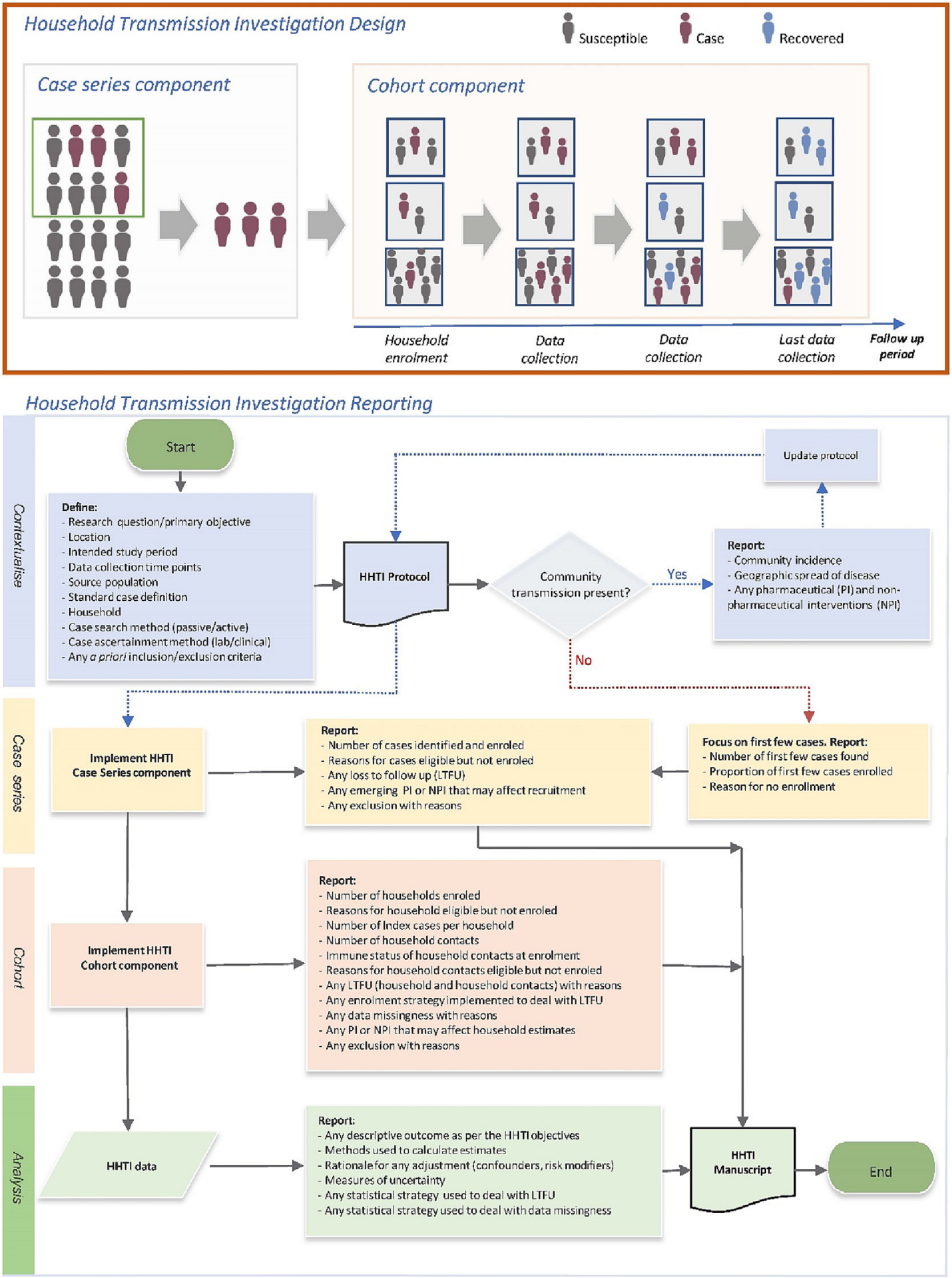
Household transmission investigation (HHTI) design and reporting. *Design:* HHTI have a case series and cohort component; cases are ascertained from a source population (design; green square) within the target population and then households are enrolled and followed‐up. *Reporting*: The adequate contextualisation of HHTI and detailed reporting of key elements of the case series, cohort and analysis steps are essential to critically appraise these investigations.

HHTIs are prospective case‐ascertained studies that investigate confirmed cases and their household close contacts.^4^ The HHTI design has two observational components (Figure 1): the identification of eligible cases from a source population (case series) and subsequent recruitment of all their household contacts on the basis of exposure to the primary case within the household (a cohort study of households).

In general, observational epidemiologic investigations seek to recruit units (people, households, etc.), to generate a sample that represents the attributes and associations present in the source population. When this principle is satisfied, the study has *internal validity* ^12^ and inferences about attributes and associations in the source population are appropriate. The study has *external validity* if such inferences can be extended to the target population from which the source population was identified.^12^, ^13^

The characteristics of populations recruited into HHTIs may differ across settings. Hence, care needs to be taken when applying learnings to different contexts. In the following, we discuss elements of HHTIs that require careful interpretation.

### 2.1 Case series component

This section discusses some key considerations of the internal validity of an HHTI, which is primarily determined by the case series component of the study. We discuss the adequate characterisation of the source population, eligibility criteria for the cases and a case definition.

#### 2.1.1 Characterisation of the source population

By design, a case series enrols participants with a specific outcome.^10^, ^14^ In the early stages of a pandemic, cases are usually sourced from populations of public health interest (e.g., returning travellers). Consequently, the attributes, associations and the disease dynamics observed in cases and their households provide epidemiologic insight but limited information about the general (target) population. Similarly, investigators conducting HHTIs in settings with established community transmission may assume that cases recruited are representative of the target population. However, cases enrolled are most likely ascertained from a specific source population (e.g., people recruited from a subset of hospitals) and even from a *secondary study base population* that is a subgroup of the source population^12^ (e.g., people recruited from a subset of hospitals with private health insurance). As these samples may not resemble the characteristics of the source and target populations, careful assessment of the internal and external validity of estimates is necessary.

#### 2.1.2 Eligibility criteria for the cases

HHTIs may focus on questions that require recruitment of cases with a specific exposure,^14^ which constrains the internal and external validity of the investigation. For example, cases may only be recruited if infected with a pathogen subtype or strain, or infected post‐vaccination. Investigations may also inadvertently recruit cases with an unmeasured exposure that could also affect the validity of the investigation, for example, a HHTI that conveniently recruits a high proportion of chronically ill patients who may have a higher risk of severe outcomes or propensity for seeking healthcare than the source population. Such awareness and understanding of disease risk among these individuals may modify estimates obtained in HHTIs.

#### 2.1.3 Case definition

Case enrolment should follow the disease's case definition with explicit considerations of the timeline over which the disease progresses and transmission can occur. In general, highly accurate diagnostic tests are preferred for case identification (e.g., Real‐Time Polymerase Chain Reaction [RT‐PCR]), though such tests may not be accessible or available, especially during the early stages of an epidemic. Where ascertainment is based on presence of symptoms, misclassification bias is likely present, particularly when syndromic criteria (e.g., acute respiratory infection or influenza‐like illness) are used as a proxy for infection with a specific respiratory pathogen (e.g., influenza or SARS‐CoV‐2). For example, attack rates may be biassed—up or down—when identification of cases is based only on syndromic criteria in the presence of multiple circulating pathogens with similar clinical presentations or a high asymptomatic fraction.

### 2.2 Cohort component

The cohort component of HHTIs aligns with the traditional concept of cohort studies, as the epidemiological units (households) are enrolled based on their exposure status, that is, the presence of a confirmed case in the household.^13^, ^14^ We highlight three aspects requiring detailed consideration: community incidence of infection; loss to follow‐up of households or householders; methods and timing of data collection.

#### 2.2.1 Community incidence

Typically, the objective of HHTIs is to rapidly generate evidence in the early stages of an epidemic to inform response activities. As a result, timely HHTI conduct increases confidence that infections in the household are not a result of exposure in the community. This is particularly important when estimating attack rates. Alternatively, HHTIs may be conducted in the later epidemic stages, for example, upon the emergence of a novel variant with differing characteristics. In each case, information generated by genomics and/or other diagnostics (e.g., serology) may assist with disentangling the effect of multiple sources of infection. Although not always accessible or available, enhanced data and analytics can provide finer resolution of transmission generations (secondary, tertiary, etc.) within the household.^15^

#### 2.2.2 Methods and timing for data collection

The duration of follow‐up must be sufficient to observe the primary outcomes. Application of diagnostic methods and associated sampling schedules must be based on the known characteristics of the pathogen and host immune response. For example, PCR should be considered to confirm active infection and/or shedding of the pathogen. Serology can be used to identify both existing immunity at recruitment and determine false negative primary diagnoses at the end of follow‐up. Sampling regimes may vary across settings due to local guidelines, resources, staffing and laboratory capacity, resulting in measurement bias.

#### 2.2.3 Participation and follow‐up

Incomplete participation and follow‐up of participants is expected in observational studies.^12^, ^13^ Random household loss‐to‐follow‐up will produce less precise epidemiologic estimates (due to smaller sample size). Random loss‐to‐follow‐up may generate different types of missing data^16^: data missing completely at random (e.g., when samples taken in a household are damaged in the laboratory) or missing at random (e.g., when samples cannot be obtained on the day of testing due to the severe course of disease of the participant). Non‐random loss‐to‐follow‐up of either individuals or households will reduce precision, accuracy, internal‐ and external‐validity. This type of loss‐to‐follow‐up results in data missing not at random (e.g., when individuals or households do not consent to be tested due to fears of stigmatisation).

## 3 REPORTING OF HHTIS

There are no published guidelines for the appropriate reporting of HHTIs, although principles from the STROBE^17^ and other guidelines^18^ are relevant. As HHTIs are frequently conducted in varied and dynamic settings, investigations conducted in different settings may capture unique phases of an epidemic, public health policies and behavioural factors (Figure 1).

Some examples of context‐specific nuances include:

- the extent of community transmission, which may be widespread, can impact confidence of the source of infection for subsequent cases and overestimate attack rates;
- non‐pharmaceutical interventions, such as mandatory isolation of cases, may remove the source of infection within a household and thus reduce estimates of attack rates or may increase time spent at home and thus household attack rates;
- pharmaceutical interventions, such as vaccination or antivirals, may reduce population susceptibility and/or reduce transmissibility, thus altering estimates of attack rates or severity;
- cultural considerations, such as the definition, size and structure of a household, can dictate inclusion criteria.

Detailed description of the local epidemiology and context in which the HHTI was implemented will enable better assessment of internal and external validity, comparison and aggregation of data across settings.

## 4 THE HHTI CRITICAL APPRAISAL CHECKLIST

Evidence synthesis is essential to inform policy and practice in health. Methodological guidelines for systematic reviews and meta‐analyses are broadly available and ensure a body of high‐quality, reproducible research.^19^, ^20^, ^21^ The critical appraisal of investigations remains a major component of systematic reviews and several appraisal tools are available.^10^, ^11^, ^22^

Despite the increasing contribution of HHTIs to inform public health policy, there is no tool dedicated to the critical appraisal of these investigations. During the COVID‐19 pandemic in the absence of a fit‐for‐purpose critical appraisal tool, generic tools for longitudinal studies,^10^, ^11^ which do not adequately capture the nuances of HHTIs, were used or partially adapted.^9^, ^23^

To address this gap, we propose a novel HHTI critical appraisal checklist, considering key elements of HHTI design, implementation and reporting.

The checklist extends on work between the WHO Unity team and a team of epidemiologists, statisticians and clinicians at The University of Melbourne who provided expert support in analysis, interpretation and reporting of HHTI data to UNITY collaborators globally. The checklist was developed to assist with the critical appraisal of original investigations aligned with the WHO UNITY protocols.^8^ The development process builds upon well‐established approaches to perform critical appraisal and risk of bias assessment of observational studies^11^, ^20^ and involved several rounds of extensive peer‐review.

Considering the reporting aspects highlighted in Figure 1, the HHTI critical appraisal checklist consists of 12 questions that broadly capture; context (Q1), index case identification (Q2), household definition (Q3), case and household recruitment (Q4), case ascertainment and subsequent case definitions (Q5), follow‐up of households (Q6a), follow‐up of individuals (Q6b), classification—susceptibility (Q7a), classification—household transmission (Q7b), analysis—methods (Q8), analysis—loss‐to‐follow‐up (Q9), and analysis—incomplete data (Q10). A rationale for each checklist component can be found in Data S1. The checklist form is in Data S2.

The checklist provides a framework to critically assess the methodological quality of HHTIs and how investigators addressed potential sources of bias in the design, conduct and analysis phases. The checklist items do not have any predetermined weighting as the importance of each checklist item should be considered in relation to the specific research question of the HHTI.

In the context of systematic reviews, all studies that meet the inclusion criteria should be appraised using this checklist by at least two investigators. Considering their objectives and the checklist outputs, investigators can make an overall assessment of the quality of reported estimates and their suitability to contribute to pooled estimates.

## 5 A PRACTICAL EXAMPLE

**FIGURE 2:**
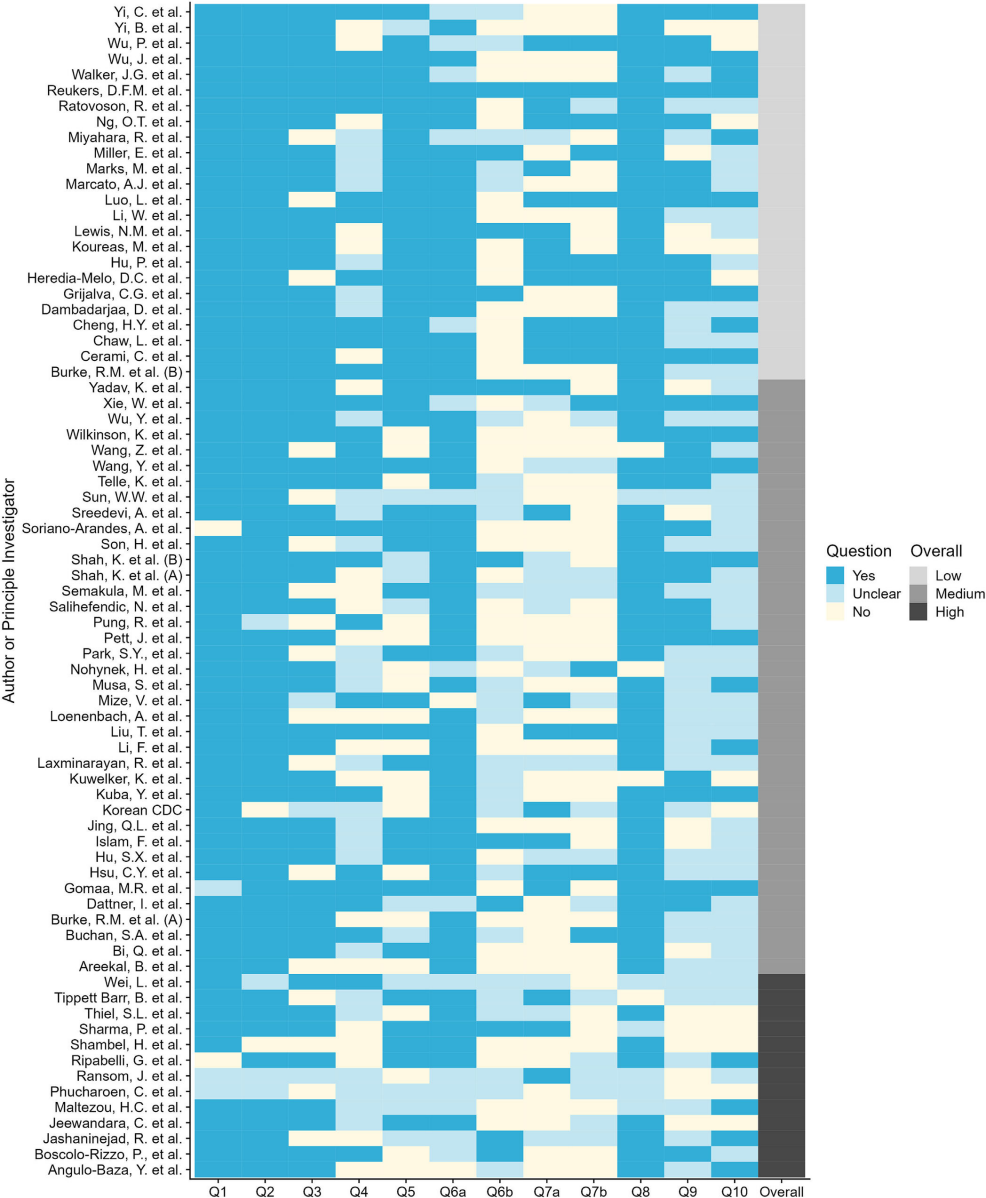
A practical example. In the transmission of SARS‐CoV‐2 in standardised first few X cases and household transmission investigations: a systematic review and meta‐analysis,^8^ teams of two investigators critically appraised a total 80 studies eligible to be included in the systematic review.

Lewis et al.^8^ conducted a systematic review of the household secondary attack rate (hSAR) of SARS‐CoV‐2 from investigations aligned with the WHO Unity Studies HHTIs protocol. Investigations included in the systematic review were assessed using the HHTI Critical Appraisal Checklist. Emphasis was placed on the definition of the household (Q3), secondary case ascertainment (Q5) and duration of follow‐up of household members (Q6b) to indicate potential for bias and thus their suitability to contribute towards aggregated estimates of hSAR (Figure 2).

Results of the critical appraisal checklist as applied to investigations that reported household secondary infection attack rate (hSAR). Colours for Questions 1–10 indicate whether each was addressed in the investigation (dark blue) or not (cream), or instances where there was insufficient detail available to assess (light blue). An overall rating of the risk of bias is provided in the far‐right column, with investigations rated Low (light grey), Medium (medium grey) or High (dark grey).

## 6 CONCLUSION

HHTIs generate critical evidence to inform policy and public health action.

Throughout the COVID‐19 pandemic, HHTIs have generated highly variable evidence across regions where interpretation is dependent on the often‐unreported local context.^7^ Inadequate appraisal of these sources of variability, including the context, design and implementation, impedes timely and fair comparisons of epidemiologic estimates generated by HHTIs. This manuscript has discussed key aspects of the HHTI design, provided recommendations for their reporting and proposed a checklist to assist their design and critical appraisal. This manuscript fills a gap in the epidemiologic literature and aims to standardise HHTI approaches to produce richer and more informative evidence to support policy.

We encourage those conducting HHTIs to consider this checklist at the design stage to ensure rigorous implementation and reporting.

## AUTHOR CONTRIBUTIONS

**David Price**: Conceptualization; formal analysis; investigation; methodology; writing—original draft, writing—review and editing. **Violeta Spirkoska**: Conceptualization; formal analysis; investigation; methodology; writing—original; writing—review and editing. **Adrian Marcato**: Conceptualization; formal analysis, investigation, methodology, writing—original draft, writing—review and editing.

**Niamh Meagher**: Conceptualization; formal analysis; investigation; methodology; writing—original draft; writing—review and editing. **James Edward Fielding**: Formal analysis; investigation; methodology; writing—review and editing. **Amalia Karahalios**: Formal analysis; investigation; methodology; writing—review and editing. **Isabel Bergeri**: Formal analysis; investigation; methodology; writing—review and editing. **Hannah Lewis**: Formal analysis; investigation; methodology; writing—review and editing. **Marta Valenciano**: Formal analysis; investigation; methodology; writing—review and editing. **Richard Pebody**: Formal analysis; investigation; methodology; writing—review and editing. **Jodie McVernon**: Formal analysis; investigation; methodology; writing—review and editing. **Juan Pablo Villanueva‐Cabezas**: Conceptualization; formal analysis; investigation; methodology; project administration; supervision; writing—original draft; writing—review and editing.

## CONFLICT OF INTEREST STATEMENT

The authors declare no conflicts of interest.

## Supporting information


**Data S1** Rationale for checklist components.

**Data S2** HHTI Critical Appraisal Checklist.

## Data Availability

No data was generated in this work.
