# Supplementary material for: Household transmission investigation: Design, reporting and critical appraisal
Source: Influenza Other Respir Viruses. 2023 Jun 15;17(6):e13165. doi: 10.1111/irv.13165 (PMC10271595; doi:10.1111/irv.13165)
Supplement: Supplementary file 1 — Data S1 Rationale for checklist components. [file IRV-17-e13165-s002.docx]

**Annex 1.** Rationale for checklist components

Household Transmission Investigation: Design, Reporting and Critical Appraisal.

David J Price^1,2^; Violeta Spirkoska^1,3^; Adrian J Marcato^1^; Niamh Meagher^1^; James E Fielding^1,3^; Amalia Karahalios^2^; Isabel Bergeri^4^; Hannah Lewis^4^; Marta Valenciano^4,5^; Richard Pebody^6^; Jodie McVernon^1,3^; Juan-Pablo Villanueva-Cabezas^1,7*^

^1^ Department of Infectious Diseases, The University of Melbourne, at the Peter Doherty Institute for Infection and Immunity, Victoria, Australia

^2^ Centre for Epidemiology & Biostatistics, Melbourne School of Population & Global Health, The University of Melbourne, Melbourne, Victoria, Australia

^3^ Victorian Infectious Diseases Reference Laboratory Epidemiology Unit, Royal Melbourne Hospital, at the Peter Doherty Institute for Infection and Immunity, Victoria, Australia

^4^ World Health Organization, Geneva, Switzerland

^5^ Epiconcept, Paris, France

^6^ World Health Organization Regional Office for Europe, Copenhagen, Denmark

^7^ The Nossal Institute for Global Health, The University of Melbourne, VIC 3000, Australia

Following the definitions in the WHO Unity protocols, a primary case refers to the first person to bring the infection into the household, and subsequent cases are secondary, tertiary, quaternary, etc., infected within the household. Index case refers to the first case reported or found in a household (thus leading to the identification of the household for recruitment into the HHTI). Following an investigation, index cases may be classified as a primary, co-primary or secondary case.

***Q1. Was the timing of recruitment and data collection appropriate to achieve the objectives of the investigation?***

To understand and contextualise the estimates reported, the investigation should describe:

- the research question appropriate to context;
- setting and context (e.g., application of non-pharmaceutical measures to constrain transmission and/or pharmaceutical interventions including vaccines, demographics);
- the epidemiologic situation (e.g., community incidence);
- how cases were identified (e.g., through active or passive surveillance);
- how cases and contacts were managed, if this is likely to influence transmission in household environments

**Rationale**. HHTI can provide high-quality epidemiologic estimates when conducted in the early stages of an outbreak, although case ascertainment may be biased towards detection of severe cases if there is over-reliance on passive surveillance. With increasing community transmission and improved understanding of the spectrum and severity of disease, there is increased sensitivity of surveillance systems but also increased likelihood of household transmission being influenced by community infection. To allow for an adequate assessment of these factors, the investigation should clearly describe the collective assessment of the epidemiologic context (i.e., community incidence), surveillance method for identification of first cases (e.g., through active or passive surveillance), any relevant public health policy and changes in population behaviour that may influence the design, conduct and outputs of the investigation.

***Q2. Was the method for index* *case ascertainment appropriate?***

Investigators should report:

- the standard case definition being used for index case ascertainment including epidemiological, laboratory, clinical and/or medical criteria;
- any relevant index case eligibility criteria.

**Rationale**. Index cases should be identified using a case definition that includes epidemiological, laboratory, clinical and/or medical criteria specific to the pathogen of interest, the context of the investigation and the availability of resources. Investigators should report if case ascertainment is conducted among a population with specific characteristics or exposures. The lack of a case definition undermines the internal validity of the HHTI.

***Q3. Was a definition of ‘household’ provided?***

To correctly interpret and compare the estimates reported, the investigators should report:

- a comprehensive definition of household that clearly captures local cultural, social, and political context;
- any relevant household eligibility criteria.

**Rationale**. The household is the main epidemiologic unit of interest. The definition of household may vary across settings estimates obtained in HHTIs can vary widely. For example, households can be defined based on:

1. groups of people living together with or without a specified minimum or maximum number of people;
2. The extent of contact (duration and type of contact) including visitors, extended family, etc.;
3. Number of dwellings occupied by the householders;
4. Sharing common areas such as cooking areas;
5. Sharing of meals (per unit of time);
6. Other social and cultural characteristics.

Investigations should report a comprehensive definition that clearly captures the local understanding of the household and makes explicit how the primary case and the household close contacts are part of it. Investigators should report if households are selected based on specific characteristics (e.g., the presence of a child within the household).

***Q4. Were all eligible cases and all householders enrolled into the investigation?***

Investigators should provide a clear description of the:

- recruitment process;
- the number of cases and household contacts eligible and enrolled;
- the number of cases and household contacts eligible and not enrolled, and why.

**Rationale**. The study should provide details about the screening and recruitment processes, where possible including the number of cases and households eligible that were and were not enrolled, as well as providing reasons.

Investigations that intend to provide early epidemiologic estimates should report the number of first few cases identified and their success enrolling them.

***Q5. Were subsequent cases identified and ascertained using appropriate methods?***

The investigators should describe how subsequent cases in the household were ascertained by detailing:

- a definition for primary, co-primary, secondary, tertiary cases with relevant epidemiological, clinical and laboratory criteria;
- methods for subsequent case ascertainment;
- any variation in subsequent case ascertainment according to study timing, context of data collection, and resource constraints.

**Rationale**. The ascertainment of subsequent cases (e.g. primary, secondary, etc.) should be based on a standard case definition appropriate to the timing, context of data collection, and resource constraints. For example, case ascertainment for COVID-19 is usually based on laboratory confirmation, regardless of symptoms (i.e., nucleic acid testing such as RT-PCR, cell culture with confirmatory nucleic acid testing, genomic sequencing or immunoglobulin assays that detect a four-fold seroconversion in acute and convalescent sera); however, these methods may not be available across settings. A combination of laboratory and symptom data will enhance ascertainment of subsequent cases as well as helping accurately classify household members, leading to greater insight into severity measures/outcomes.

***Q6a. Was the duration of follow-up sufficient to measure outcomes?***

Investigators should report how long households were followed-up and:

- describe the relevance of follow-up duration to measure the outcomes in relation to known epidemiological parameters.

**Rationale**. The duration of household follow up should be determined in relation to key epidemiological parameters of the pathogen of interest, the testing methods available, and outcomes. The timing of sampling should be appropriate for the specified methods. For example, RT-PCR typically has increased sensitivity during the earlier stages of infection, whilst serology is more likely to be effective at identifying an infection during later stages of an infection when antibodies are produced.

***Q6b. Did all participants remain part of the ‘household’ for the duration of the investigation?***

Investigators should clearly report with reasons:

- removal of participants from the household during the investigation;
- any hospitalisation of cases;
- any other loss-to-follow-up (household, individual, or both).

**Rationale**. The primary case and the household contacts should remain part of the household for the duration of the study. Removal of cases from the household at enrolment, or during the investigation (e.g., admitted to hospital or deceased), should be clearly reported. Removal of cases may affect the degree of exposure of household contacts to the pathogen, thus potentially biasing the epidemiologic estimates obtained. Similarly, the removal or loss to follow up of household contacts (cases and not cases) may lead to biased epidemiologic estimates and therefore should be clearly reported.

***Q7a. Were steps taken to characterise the susceptibility of the householders at the time of enrolment?***

To determine the extent of susceptibility, investigators should clearly report:

- information relating to the immune status of participants at enrolment (susceptible, infected, infectious, sero-positive) and throughout the investigation;
- their confidence of the classification of individuals.

**Rationale**. HHTI should enrol households in which the primary case meets the case definition (Q2) and householders are susceptible to infection. To facilitate the assessment of the epidemiologic estimates, the investigation should ascertain the immune status of participants at enrolment (e.g., susceptible, infectious, sero-positive) and sensitivity and specificity of the method. In the context of community transmission and/or the commencement of vaccination campaigns, baseline serological samples are recommended to account for past infection or vaccination, and to determine the extent of susceptibility.

***Q7b. Were steps taken to identify the source of subsequent infections within the household?***

To assess transmission within the household, investigators should clearly report:

- whether cases remain in the household
- quarantine, isolation and mitigation measures within the household
- community incidence.

**Rationale**. Investigators should ensure that the estimates obtained and reported are the product of disease dynamics occurring within the household. These dynamics are influenced by the presence of cases within the household, quarantine and isolation measures occurring within the dwelling or outside it (e.g., when cases are isolated in hospitals) and by the community incidence. Where co-primary cases are identified and included, investigators should provide evidence that each co-primary case acquired the infection independently. Whole genome sequencing can provide insight into transmission chains and confirm infections within the household. Unfortunately, genomics is beyond the scope of most HHTIs. In the absence of more complex methods, analyses that explore the sensitivity to classification of co-primary cases should be conducted and reported.

***Q8. Are the analytic methods appropriate given the study context and design?***

To interpret the estimates derived from the HHTI, investigators should:

- report detailed methods used to calculate the estimates including the rationale for any adjustments made (e.g., confounders, risk modifiers);
- provide measures of uncertainty for the estimates reported;
- provide appropriate interpretation and limitations of the results;
- assess how robust their estimates are to changes in classification, where appropriate.

**Rationale**. Investigators should attempt to follow the “Statistical analysis plan for First Few X cases and contact investigations, Household Transmission investigations and Closed settings investigations protocol”^6^. The statistical methods used to generate crude and adjusted estimates should be described to facilitate the correct interpretation and reproducibility where data are available. Crude and adjusted estimates should be accompanied by an appropriate measure of uncertainty. Any assumptions made should be reported.

***Q9. Has loss-to-follow-up been appropriately accounted for in the estimated outcomes?***

If loss-to-follow-up exists (Q6b), investigators should:

- describe how loss-to-follow-up was accounted for (household, individual, or both) including any assumptions made.

**Rationale**. Random and systematic loss to follow up have different effects on the epidemiologic estimates produced in HHTIs. The investigators should attempt to characterise the households and/or participants that did not remain in the study for the whole period of investigation. If loss to follow up was accounted for (e.g., replacement of households, elimination of incomplete households), the study should clearly report the approach, the rationale for it, and any assumptions made.

***Q10. Has any missing data been appropriately accounted for in the estimated outcomes?***

Where data is incomplete, investigators should:

- describe the proportion of missing data for all relevant variables;
- report reasons for missing data;
- report how missing data were accounted for, including assumptions made.

**Rationale**. Households or participants may not provide all of the required data, which may affect the epidemiologic estimates produced. Investigators should report the proportion of missing data and whether these are missing completely at random, missing at random, or missing not at random especially when variables are relevant for the primary outcomes (e.g., presence of symptoms). If missing data was accounted for (e.g., imputation, retrospective data collection), investigators should report the approach, rationale, and assumptions made.
