# Supplementary material for: Household transmission investigation: Design, reporting and critical appraisal
Source: Influenza Other Respir Viruses. 2023 Jun 15;17(6):e13165. doi: 10.1111/irv.13165 (PMC10271595; doi:10.1111/irv.13165)
Supplement: Supplementary file 2 — Data S2 HHTI Critical Appraisal Checklist. [file IRV-17-e13165-s001.docx]

**Annex 2.** HHTI Critical Appraisal Checklist

Household Transmission Investigation: Design, Reporting and Critical Appraisal.

David J Price^1,2^; Violeta Spirkoska^1,3^; Adrian J Marcato^1^; Niamh Meagher^1^; James E Fielding^1,3^; Amalia Karahalios^2^; Isabel Bergeri^4^; Hannah Lewis^4^; Marta Valenciano^4,5^; Richard Pebody^6^; Jodie McVernon^1,3^; Juan-Pablo Villanueva-Cabezas^1,7*^

^1^ Department of Infectious Diseases, The University of Melbourne, at the Peter Doherty Institute for Infection and Immunity, Victoria, Australia

^2^ Centre for Epidemiology & Biostatistics, Melbourne School of Population & Global Health, The University of Melbourne, Melbourne, Victoria, Australia

^3^ Victorian Infectious Diseases Reference Laboratory Epidemiology Unit, Royal Melbourne Hospital, at the Peter Doherty Institute for Infection and Immunity, Victoria, Australia

^4^ World Health Organization, Geneva, Switzerland

^5^ Epiconcept, Paris, France

^6^ World Health Organization Regional Office for Europe, Copenhagen, Denmark

^7^ The Nossal Institute for Global Health, The University of Melbourne, VIC 3000, Australia

Date:

| **Overall Appraisal - Risk of bias** | ***Low*** | ***Medium*** | ***High*** |
| --- | --- | --- | --- |
| Comments: |  | | |
|  |  |  |  |
| **Decision** | ***Include*** | ***Exclude*** | ***Unclear*** |
| Comments: |  | | |

Investigation ID:
Author and Year:
Reviewer:

| Questions | Yes | No | Unclear | Comments |
| --- | --- | --- | --- | --- |
| 1. Was the timing of recruitment and data collection appropriate to achieve the objectives of the investigation? |  |  |  |  |
| 2. Was the method for index case ascertainment appropriate? |  |  |  |  |
| 3. Was a definition of ‘household’ provided? |  |  |  |  |
| 4. Were all eligible cases and all householders enrolled into the investigation? |  |  |  |  |
| 5. Were subsequent cases identified and ascertained using appropriate methods? |  |  |  |  |
| 6a. Was the duration of follow-up sufficient to measure outcomes? |  |  |  |  |
| 6b. Did all participants remain part of the ‘household’ for the duration of the investigation? |  |  |  |  |
| 7a. Were steps taken to ensure that householders were susceptible at the time of enrolment? |  |  |  |  |
| 7b. Were steps taken to ensure that subsequent infections were due to exposure within the household? |  |  |  |  |
| 8. Are the analytic methods appropriate given the study context and design? |  |  |  |  |
| 9. Has loss-to-follow-up been appropriately accounted for in the estimated outcomes? |  |  |  |  |
| 10. Has any missing data been appropriately accounted for in the estimated outcomes? |  |  |  |  |
